# The Application of Bicarbonate Recovers the Chemical-Physical Properties of Airway Surface Liquid in Cystic Fibrosis Epithelia Models

**DOI:** 10.3390/biology10040278

**Published:** 2021-03-29

**Authors:** Loretta Ferrera, Valeria Capurro, Livia Delpiano, Ambra Gianotti, Oscar Moran

**Affiliations:** 1 U.O.C. Genetica Medica, Istituto G. Gaslini, Via G. Gaslini, 5, 16148 Genoa, Italy; Loretta.Ferrera@unige.it (L.F.); valeriacapurro@yahoo.it (V.C.); l.delpiano2@ncl.ac.uk (L.D.); ambrygianotti@hotmail.com (A.G.); 2 Istituto di Biofisica, CNR, Via De Marini, 6, 16149 Genoa, Italy

**Keywords:** cystic fibrosis, bicarbonate, mucus, micro-rheology, multiple particle tracking

## Abstract

**Simple Summary:**

Cystic fibrosis (CF) is a multi-organ disease that affects the epithelia and exocrine glands, particularly the lungs, but also the pancreas, liver, kidneys, and intestines. Respiratory disease is the most common cause of death. Many of the problems in respiratory disease can be attributed to the viscous nature of the mucus. Indeed, thick mucus in the airways leads to reduced mucociliary clearance, chronic bacterial infections and inflammation followed by destruction of the lung parenchyma. The composition of the periciliary fluid (ASL) is regulated by the transport of water and ions through different ion channels and transporters distributed in a non-symmetrical way to the two parts of the epithelium. Mucus is the first line of defense against inhaled particles and protects the epithelium by trapping and eliminating harmful substances through the blink of the cells that push them towards the nose. In patients with CF the malfunction of the mutated CFTR causes a reduction in bicarbonate secretion with consequent reduction in the expansion of mucins and the formation of thick mucus. In this work we have shown that, in vitro, the administration of a solution containing bicarbonate ion would act not only on the osmotic component of the mucus, but directly on the expansion of the mucins by acting on its viscoelastic properties.

**Abstract:**

Cystic fibrosis (CF) is a genetic disease associated with the defective function of the cystic fibrosis transmembrane conductance regulator (CFTR) protein that causes obstructive disease and chronic bacterial infections in airway epithelia. Deletion of phenylalanine at position 508, p.F508del, the most frequent mutation among CF patients, causes a folding and traffic defect, resulting in a dramatic reduction in the CFTR expression. To investigate whether the direct application of bicarbonate could modify the properties of the airway surface liquid (ASL), we measured the micro-viscosity, fluid transport and pH of human bronchial epithelial cells monolayers. We have demonstrated that the treatment of a CF-epithelia with an iso-osmotic solution containing bicarbonate is capable of reducing both, the ASL viscosity and the apical fluid re-absorption. We suggest the possibility of design a supportive treatment based on topical application of bicarbonate, or any other alkaline buffer.

## 1. Introduction

Cystic fibrosis (CF) is a monogenic disease caused by the lack of function of the cystic fibrosis transmembrane conductance regulator protein (CFTR), an anion-permeable channel involved in fluid transport through epithelia [1]. Almost 2000 mutations of the CFTR gene have been identified (http://www.genet.sickkids.on.ca; accessed on 1 March 2021), and have been grouped into six mutation classes, classified according to the effect of the mutation [2,3]: failure to synthesize the protein, processing flaws, gating or conductance defects, reduced expression. Depending on their severity, these mutations prevent or reduce the secretion of ions through the epithelium. The deletion of a phenylalanine in position 508 is the most frequent mutation in CF patients (p.F508del, class II mutation, prevalence of approximately 80%). In individuals with the p.F508del mutation CFTR protein is translated, but its folding, trafficking and the stability in the membrane are altered and most of the protein is degraded inside the cell [1,2,4]. CF is a multi-organ disease affecting the epithelia and exocrine glands, mainly in the lungs, but also in the pancreas, liver, kidneys, and intestine. The CF respiratory disease is the “leading” cause of death and the disease’s primary burden on quality of life. Most of the health problems in CF respiratory disease can be attributed to the viscous mucus phenotype [5,6]. In fact, in CF a viscous and tenacious sticky mucus in the airway surface liquid (ASL) leads to inappropriate mucociliary clearance, chronic bacterial infection and inflammation, and at long last to the destruction of the lung parenchyma [2,7].

As said before, most people with CF carry the p.F508del mutation. Therefore, as they would benefit from traffic corrector compounds, a huge effort has been devoted in the last fifteen years to identifying drugs that can recover the function of mutated CFTR [8,9]. This work resulted in the discovery of an effective compound, lumacaftor (VX-809), which was approved for use in humans in 2015 [10].

In the human small airway, the maintenance of the ASL homeostasis depends on a balanced secretion of chloride and bicarbonate and the absorption of sodium in the periciliary fluid. In CF patients, the reduced bicarbonate secretion causes a lowering of the ASL pH which is involved in the change of the epithelia equilibrium with alterations in the ciliary function, mucus formation and antimicrobial activity [11,12]. When CF human bronchial primary epithelial cells (HBEC) were treated with lumacaftor, 25% of the ion transport of p.F508del-CFTR was rescued, and, even if the rate of fluid re-absorption was not modified, this functional correction was enough to reduce significantly the ASL mucus viscosity [13]. The CF mucus is due to unpacked mucin in the ASL requiring the secretion of bicarbonate [14,15], that is accomplished by the CFTR [3,16]. Interestingly, the three-fold increase of the bicarbonate permeability of the lumacaftor-rescued p.F508del-CFTR [17,18] may account for the corrector’s the efficiency in reducing the ASL mucus viscosity.

Recently, it has been proposed that direct administration of bicarbonate in CF patients should improve the their clinical conditions, in two clinical trials (NCT03391414 and NCT00177645), comparing the use of a concentrated sodium chloride solution for inhalation with an inhaled solution of sodium bicarbonate in an attempt to decrease the thickness and viscosity of the mucus in the CF lungs and they observed a marked improvement of mucociliary clearance and lung function. Furthermore, Dobay and colleagues [19] demonstrated that, in vitro, the application of bicarbonate causes a reduction in bacterial growth and blocks the formation of the biofilm from P. aeruginosa, and the authors suggested the an aerosol inhalation therapy with HCO3- solutions can help improve respiratory hygiene in patients with cystic fibrosis and possibly other chronically infected lung diseases [19]. The inhalation of a hypertonic sodium bicarbonate solution (4.2% concentration) determined an increase in airways pH value [20]. A study on sputum samples from CF patients established that application of a sodium bicarbonate solution (100 mM) reduces its viscosity [21]. Case reports of observed significant improvement in patients with acute respiratory distress syndrome (ARDS) due to COVID-19 and maximum ventilatory support after inhalation of sodium bicarbonate [20].

The impact of the direct application of bicarbonate on the ASL mucus properties of human bronchial epithelial cells from CF-patients and non-CF subjects as already recently demonstrated on the CFBE cell line [22]. Here we set out to evaluated the properties of ASL after incubation with solutions with or without bicarbonate, applied to the apical side of bronchial cells in an air-liquid interface culture. In particular, we analyzed action of bicarbonate on the viscosity of the mucus to confirm its fluidification in treated epithelia and subsequently we also highlighted the changes of the pH of the mucus and re-absorption of the fluid after applying bicarbonate. Noteworthily, the bicarbonate treatment may be easily translated in a treatment for CF patients, representing a mutation-independent therapy to clear the airways of thickened mucus and improve lung function [13,21,23].

## 2. Materials and Methods

### 2.1. Cell Culture

The procedure for human bronchial epithelial cells (HBEC) isolation, culture and differentiation is described in detail elsewhere [24]. Briefly, HBEC were obtained from human mainstem bronchi, derived from three different F508del homozygous CF patients and three different non-CF individuals undergoing lung transplant. The collection of human cells were approved by the ethical committee of the Istituto Giannina Gaslini following the guidelines of the Italian Ministry of Health. Each patient provided informed consent to the study using a form that was also approved by the Ethics Committee.

To isolate HBEC, the bronchi were dissected, washed and incubated overnight at 4 °C in protease XIV solution, and then cultured in a serum-free proliferative medium (LHC9-RPMI 1640, 1:1) complemented with various hormones and supplements. This medium promotes the amplification of cells number [25]. To obtain differentiated epithelia, HBEC were sown at a high density on Snapwell permeable supports (12 mm Snapwell inserts, code 3801, Corning, New York, NY, USA) and after 48 h the proliferative medium was replace with DMEM/Ham’s F12 (1:1) containing 2% fetal bovine serum plus hormones and supplements added only in the basolateral side (ALI condition). This culture was maintained for 4–5 weeks before performing the experiments, in order to promote further differentiation of the epithelium. This was verified by measuring transepithelial electrical resistance and potential difference with an EVOM2 epithelial voltohmeter (WPI, Sarasota, FL, USA) before applying bicarbonate. Transepithelial resistance and potential difference of 7–8 days old epithelia were ≥1 KΩ·cm^2^ and ≈ −20 mV, respectively, confirming the correct differentiation of the epithelium [24]; the measurement of the electrical parameters of the epithelia was also done before the to the experimental procedure. To recover the mucus, the apical surface of epithelia kept in culture between 3 and 8 weeks was washed twice with a Ringer’s solution containing (in mM): 150 NaCl, 5 KCl, 1.2 CaCl_2_, 0.5 MgCl_2_ and 0.1 Hepes (pH 7.4), and incubated for 48 h. Bicarbonate at different concentrations were applied substituting NaCl by NaHCO_3_. At end of incubation, the epithelial mucus was carefully aspirated from the apical surface and obtaining a preparation suitable for evaluating the its properties. The volume of each sample was evaluated gravimetrically, assuming a specific volume of 1 µL/mg; an aliquot was used to measure the protein content with the Bradford assay. Comparison of the parameters evaluated done on fresh mucus and on the same sample kept frozen for 25 days showed no difference.

### 2.2. Micro-Rheology

The microrheological properties of the fluid recovered from the apical side of epithelia were analysed for the using the Multiple Particle Tracking (MPT) method as described [13,26]. In MPT, the time course of the position of nano-spheres in suspension inside the medium to be studied is recorded. An aliquot of 25 µL of mucus and 1 µL of solution containing carboxylated polystyrene yellow/green fluorescent beads of 200 nm in diameter (λ_ecc_ = 488 nm, λ_em_ = 505–515 nm; Fluospheres, Life Technologies, Monza, Italy) were mixed. A sample of 8 µL of mucus containing < 100 beads/field were deposited between two glass coverslips, and the borders were sealed to avoid evaporation. After 10 min equilibration at room temperature, the beads were focused to the mid-height of the sample, to exclude beads that might be interacting with the coverslips, with a 60× (N.A. = 1.42) oil immersion objective. Images were recorded with a CCD video camera (Teledyne Photometrics, Tucson, AZ, USA). Images of 1392 × 1040 pixels were captured at 6 frames/s. The trajectory of the beads were recorded using the ImageJ Multitracker plug-in [27]. About 400 beads were tracked in four to eight fields per sample.

The average movement of the fluorescent beads in a given time interval, *τ*, is described by its mean squared displacement, <msd>; the displacement of beads in viscous solutions shows a linear dependence to the time interval *τ* of the form:

(1) $$\langle \mathrm{msd}\left(\tau \right)\rangle =4D_{0}\tau^{\alpha }$$

from which it is possible to calculate *D_o_*, the diffusion coefficient. The elastic component of the fluid, as in the case of mucus, results in a non-linear *τ*-dependence of <msd>, where 0 < *α* < 1. The viscosity η was calculated from the Stokes-Einstein’s equation.

### 2.3. pH Measurement

A sample ≥ 40 µL of the fluid and mucus mixture was obtained after 48 h treatment. To minimize the effect of the diffusion of carbonic dioxide, present in the cell incubator and plausibly in equilibrium with the fluid, ASL samples were rapidly collected in the incubator, and immediately measured (within 2 min) using a microelectrode (SevenCompact, Mettler-Toledo, Novate Milanese, Italy). The measurements were repeated at least in triplicate, discarding the discordant measurements.

### 2.4. Measurement of Fluid Re-Absorption

To calculate the fluid re-absorption of the epithelia, 130 µL of Ringer’s solution was applied on the apical side of the permeable support. DMEM/Ham’s F12 culture medium was kept in the basolateral side along the experiment. After 48 h, the remaining mixture of fluid and mucus was carefully recovered and weighted. The net flux across the epithelium, *J_W_*, was calculated as:

(2) $$J_{W}=\frac{V_{i}-V_{f}}{A\times t}$$

where *V_i_* and *V_f_* are the initial and final apical volumes measured gravimetrically, *A* is the epithelium area (for Snapwell support: 1.13 cm^2^), and *t* is the time interval between addition of *V_i_* and recovering of remaining fluid *V_f_*.

### 2.5. Chemicals

For culture media and supplements, we used the materials described by Gianotti and co-workers [24]. Unless explicitly stated, all other chemical were purchased from Sigma-Aldrich (Milano, Italy).

### 2.6. Data Analysis and Statistics

All statistical and MPT analysis was done with IgorPro 8.0.4 software (WavemetricsLake, Oswego, OR, USA). Data are shown as mean ± standard error of the mean. Comparison between a data was done using Student’s *t* test, after controlling the normal distribution of data with the Shapiro-Wilk normality test. A value of *p* < 0.05 was considered statistically significant.

## 3. Results

### 3.1. Rheological Properties

**Figure 1:**
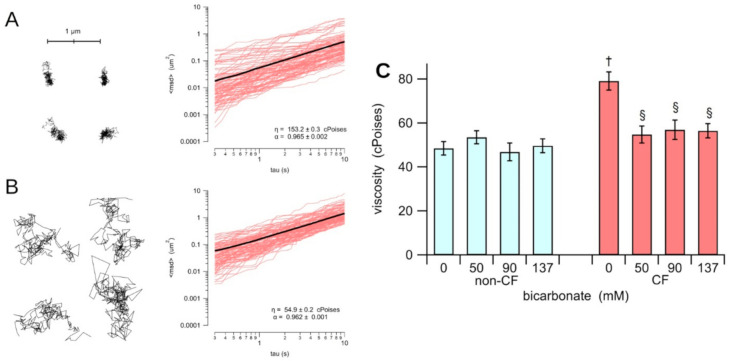
Micro-rheology of the apical fluid recovered from the HBEC-monolayers cultured on Snapwell permeable supports. The trajectory of four nanospheres were recorded in ASL from CF epithelia in a 0 bicarbonate solution (**A**) and from CF epithelia incubated with 90 mM bicarbonate (**B**). The plot of the square displacement against the time interval of 100 nanospheres are shown. The mean square displacement is indicated as a black solid line. The valued of the viscosity (η) and the no-linear parameter α are indicated in each plot. The panel (**C**) shows the average of the viscosity of the ASL collected from non-CF (cyan) and CF (red) HBEC-monolayers treated with iso-osmotic solutions with different concentrations of bicarbonate. The section sign (§) indicates that data is statistically different (*p* < 0.0007) from the CF in 0 bicarbonate solution; the dagger (†) indicates that, in 0 bicarbonate solution, CF is statistically different (*p* < 0.0001) from the non-CF.

The valuation of micro-rheology properties of the fluid recovered from the apical side of CF and non-CF epithelia has shown that, also in vitro, the viscosity of the CF HBEC ASL incubated in a 0 bicarbonate solution (79.1 ± 4.1 cPoises; n = 49), is significantly greater (*p* < 0.0001) than the viscosity measured on the ASL from the non-CF HBEC epithelia in a 0 bicarbonate solution (48.5 ± 3.0 cPoises; n = 42). Incubation of non-CF HBEC epithelia with iso-osmotic bicarbonate solutions does not produce any significant change in ASL viscosity (53.5 ± 3.0 cPoises, n = 17, 46.9 ± 4.0 cPoises, n = 15, 49.7 ± 3.1 cPoises, n = 18, for 50 mM, 90 mM and 137 mM bicarbonate, respectively; Figure 1C).

Otherwise, treatment with bicarbonate significantly reduces the viscosity of the ASL in the homozygous p.F508del. It is clearly seen in the increase of the displacement of the fluorescent nanospheres when comparing the trajectory observed in the mutant-CFTR epithelium in 0 bicarbonate (Figure 1A) with the trajectory observed in the same mutant-CFTR epithelia treated with 90 mM bicarbonate (Figure 1B). Figure 1A,B show that the viscosity of the ASL calculated from the plot of the mean square displacement against the time interval results in a significantly reduced (*p* < 0.0007) CF HBEC monolayers treated with bicarbonate, yielding 54.8 ± 3.9 cPoises, n = 18, 56.9 ± 4.4 cPoises, n = 16, 56.4 ± 3.3 cPoises, n = 24, for 50 mM, 90 mM and 137 mM bicarbonate, respectively. Notice that the ASL of the non-CF HBEC epithelia treated with iso-osmotic bicarbonate solutions have a viscosity that is not statistically different to that measured in control non-CF epithelia.

The elastic component of the ASL is expressed by the coefficient α, which represents the non-linearity of the mean square displacement of the nanospheres in the MTP experiments. The value of α of ASL from non-CF HBEC (0.95 ± 0.01; n = 29) is not statistically different from that of the CF HBEC (0.95 ± 0.01: n = 26). The bicarbonate treatment does causes any significant change of α, neither on ASL of the non-CF HBEC (0.96 ± 0.01; n = 15), nor on the p.F508del ASL (0.96 ± 0.01; n = 13).

To estimate the amount of mucin released by cells, we have measured the amount of protein in the ASL, assuming that most of it is mucin. The amount of protein in the fluid of the non-CF HBEC (49.4 ± 5.5 mg; n = 22), which is not significantly different from the amount of protein in the ASL of the CF epithelia (56.5 ± 5.8 mg; n = 24). Incubation of CF HBEC epithelia with 90 mM bicarbonate does not significantly change the amount of ASL proteins (62.8 ± 7.6 mg; n = 14). Therefore, the substantial difference between the viscosity of the CF HBEC ASL and the viscosity of the non-CF HBEC epithelia incubated in a 0 bicarbonate solution could be correlated with the more acid pH in the CF epithelia, which reduces the post-secretion expansion of the ASL mucins.

### 3.2. The ASL pH

Thus we decided to evaluate the pH of the apical fluid. The pH was measured in ASL collected after bicarbonate treatment with a pH microelectrode. Even if we take the necessary precautions to minimize the diffusion of carbonic dioxide from the fluid, our measurements are biased, probably being slightly alkaline. However, the pH values obtained accurately reflect the conditions of the epithelium and the treatment to which they were subjected. 

**Figure 2:**
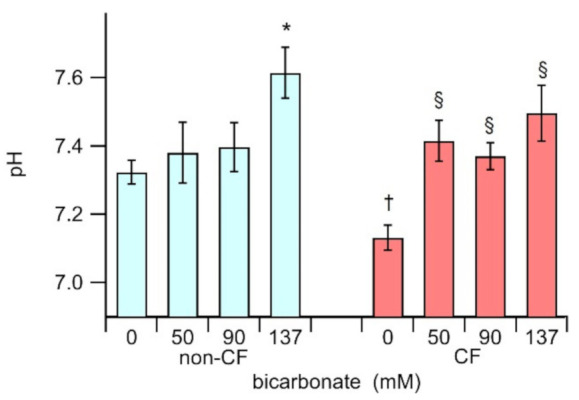
pH of the apical fluid recovered from the HBEC-monolayers, cultured on Snapwell permeable supports, from three different non-CF (cyan), and three different CF (red) subjects, treated with iso-osmotic solutions with different concentrations of bicarbonate. The asterisk (*) indicates that data is statistically different (*p* < 0.03) from the non-CF incubated in 0 bicarbonate solution; the section sign (§) indicates that data is statistically different (*p* < 0.0006) from the CF in 0 bicarbonate, and the dagger (†) indicates that untreated CF is statistically different (*p* < 0.0005) from the non-CF in 0 bicarbonate.

Data presented in Figure 2 shows that ASL from CF epithelium incubated in 0 bicarbonate 0 (7.13 ± 0.04, n = 30) is significantly more acidic (*p* < 0.0001) than ASL from non-CF under the same conditions (7.32 ± 0.04 n = 18). As expected, incubation of the homozygous p.F508del epithelium with bicarbonate on the apical side causes a significant alkalinisation of the ASL (*p* < 0.0006), yielding a pH of 7.42 ± 0.08 (n = 10), 7.37 ± 0.04 (n = 30), and 7.50 ± 0.08 (n = 13) for HBEC monolayers treated with 50 mM, 90 mM and 137 mM bicarbonate, respectively. Conversely, in non-CF epithelia, the incubation with 50 mM and 90 mM bicarbonate did not significantly modify the ASL pH (7.38 ± 0.09, n = 6; 7.39 ± 0.07, n = 17, respectively), but was required to incubate the cells with a higher bicarbonate concentration of 137 mM, to alkalinize the ASL (7.62 ± 0.08, n = 12). We speculate that the bicarbonate does not easily modify the pH on non-CF epithelia because the presence of functional CFTR would actively participate in the regulation the ASL milieu, perhaps reabsorbing the excess bicarbonate.

### 3.3. Transepithelial Fluid Re-Absorption

**Figure 3:**
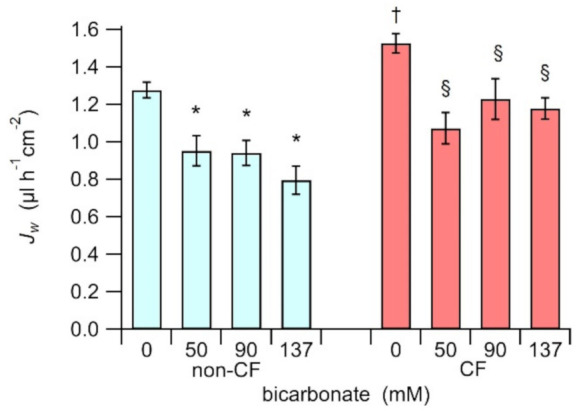
Fluid re-absorption, *J_W_*, of HBEC-monolayers, cultured on Snapwell permeable supports, from three different non-CF (cyan), and three different CF (red) subjects, treated with iso-osmotic solutions with different concentrations of bicarbonate. The asterisk (*) indicates that data is statistically different (*p* < 0.02) from the non-CF incubated with 0 bicarbonate solution; the section sign (§) indicates that data is statistically different (*p* < 0.001) from the CF treated with 0 bicarbonate solution; the dagger (†) indicates that CF and non-CF cells treated in absence of bicarbonate are statistically different (*p* < 0.0001).

When cells were incubated in the absence of bicarbonate, the fluid reabsorption of a non-CF epithelium, where the CFTR basal activity is physiological, is 1.28 ± 0.04 µL/h/cm^2^ (n = 63), while the fluid reabsorption of homozygous p.F508del-HBEC epithelia is significantly higher (*p* < 0.0001), with an average value of 1.53 ± 0.05 µL/h/cm^2^ (n = 48). This increased fluid reabsorption in CF-HBEC is consistent with a dehydrated ASL [28,29,30]. Incubation with an iso-osmotic solution with bicarbonate causes a reduction of the fluid re-absorption in both, non-CF and homozygous p.F508del epithelia. Treatment with 90 mM bicarbonate yields JW values of 0.94 ± 0.07 µL/h/cm^2^ (n = 31) and 1.23 ± 0.11 µL/h/cm^2^ (n = 26), respectively for non-CF- and CF-HBEC (Figure 3). These data show that the treatment with iso-osmotic solutions with bicarbonate reduces the fluid re-absorption.

These results indicate that the presence of an iso-osmotic solution with a relatively high concentration of bicarbonate in the apical side of the epithelia may modify some transport mechanisms, altering the ionic homeostasis and reducing the fluid re-absorption. Plausibly, this effect could contribute restoring the correct hydration of the ASL in the CF-epithelia.

## 4. Discussion

The composition of the ASL is regulated by the transport of water and ions through different ionic channels and transporters non-symmetrically distributed in the two sides of the epithelium [28,29,30,31,32]. In the airways, sodium is absorbed apically through epithelial sodium channel (ENaC), and chloride is secreted apically mainly by CFTR channels, with a passive transepithelial flow of water to regulate the ASL volume [33,34]. Another chloride channel expressed in the airway epithelium, such as the Ca2 + activated chloride channel (TMEM16A), could be modulated to replace the mutated CFTR channel. TMEM16A may improve airway mucus hydration and increase mucociliary clearance, but so far its activation by denufosol has not shown any benefit for CF patients [35]. The modulation of other mechanisms, such as purinergic receptors [36] or the bicarbonate/chloride antiporter (SLC26A9) [37] have also been suggested as targets for the treatment of CF.

On the other hand, the mucus in the ASL is the first-line defence against inhaled particles, and it protects the epithelium by entrapping the noxious substances and by removing them by the mucocilliary clearance mediated by ciliary beating [32,34]. The unpacking of mucins requires bicarbonate [14,15], which is primarily secreted by the CFTR. Thus, in the CF epithelia, the failure of the mutant CFTR to transport chloride and the lack of its inhibitory regulation of the ENaC leads to a decreased ASL volume. In this condition, mucus layer collapses up the cilia and stops mucocilliary clearance, suppressing innate immunity [32,38]. Further, the malfunction of CFTR reduces the bicarbonate secretion, crucial for mucins expansion, that is linked with the phenomenon of thick mucus [39,40].

The use of correctors have represented a considerable success on the therapy of CF patients carrying mutations with folding and trafficking defects (class II). Correctors are small molecules that are able to increase the amount of the p.F508del-CFTR protein on the plasma membrane, avoiding its retention in the endoplasmic reticulum, and its rapid degradation [1,4]. The first corrector used in patients is lumacaftor, alone or in the combination with the potentiator ivacaftor [10]. The treatment of HBEC with lumacaftor turned out in a reduction of the ASL viscosity, but without any significant benefit for the fluid re-absorption of the epithelia [13]. Differently, here we demonstrated that the treatment of a CF-epithelia with an iso-osmotic solution containing bicarbonate is capable to reduce both, the apical fluid reabsorption and the ASL viscosity. The reduction of the fluid re-absorption in CF epithelia should represent a larger ASL volume, that would improve the mucociliary clearance that is compromised in the CF pulmonary disease. The mechanism by which the application of bicarbonate leads to a decrease in fluid reabsorption remains without a clear explanation. It could be hypothesized, in particular in the CF epithelia, that the increase in pH that occurs due to bicarbonate treatment could lead to a decrease in ENaC activity [41] modifying the fluid transport. Indeed, inhibition of the sodium secretion on CF-epithelia resulted in a reduction of the fluid reabsorption [13]. Thus, we shall conclude that the lumacaftor-rescued p.F508del-CFTR is not able to transport enough anions to regulate the ionic composition of the ASL, or cannot regulate the excess sodium re-absorption due to ENaC dis-regulation [13].

Our data, presented herein, shows that in a virtual absence of CFTR in CF-epithelia a moderately high concentration of bicarbonate is enough to increase the ASL pH with the consequent reduction of mucus viscosity. As our data do not show a significant change in the protein contents of the fluid, we hypothesize this phenomenon is independent of the secretion of mucin. Intriguing, this increase of the ASL pH appears to be unrelated to the bicarbonate concentration, suggesting that, when a relatively large concentration of bicarbonate is present in the apical side of the epithelia, mechanisms other than CFTR would participate in its reabsorption. The consequence is that the mucus viscosity of CF-treated epithelia is reduced to levels similar to those of the non-CF epithelia. Conversely, the change in pH caused by concentrations ≤ 90 mM of bicarbonate does not cause a significant change in the pH of the ASL, probably because the active CFTR in non-CF cells is able to regulate the ASL homeostasis during the 48 h incubation, and therefore there are no variations in the viscosity of the ASL. Interestingly, even though application of 137 mM bicarbonate to non-CF epithelia causes a significant increase in ASL pH, we did not observed any change in viscosity. To date, the role of the ASL pH value in CF disease remains controversial [42]. In fact, Schultz and colleagues found no differences in the ASL pH obtained from children with CF compared to healthy controls [12], while Cho’s group showed a significant difference between the ASL pH of normal and CF patients [43]. In any case, a lower ASL pH, caused by reduced bicarbonate secretion, should be the potential mechanism determining lung infections and lung complications associated with CF. We can hypothesize that, in addition to some level of alkalinisation of the ASL, most of the mucus has been untangled, and therefore no effect on viscosity could be observed by further increase in pH. However, in our experimental conditions, it is impossible to discern the distinct role of bicarbonate and pH in controlling water flow and viscosity. Experiments in which the bicarbonate concentration is modified keeping the pH constant, for example by changing the CO_2_ partial pressure will serve to differentiate the role of each of these parameters.

An interesting question is why lumacaftor cannot correct p.F508del-CFTR enough to recover correct ion homoeostasis, and consequently the ASL volume control, but can reduce the ASL viscosity. One possible explanation is that the corrector-rescued CFTR, despite having a relatively low plasma membrane density and a diminished gating activity, it would exhibit an increased bicarbonate secretion. In fact, in native CFTR anion channels, the relative chloride to bicarbonate channel permeability, PHCO3/PCl, is 0.36 (it permeates about one bicarbonate ion for every three chloride ions), while in the lumacaftor-rescued p.F508del-CFTR the bicarbonate permeability is three-fold increased, yielding a PHCO3/PCl of 0.93 (the rescued mutant permeates about one bicarbonate ion for every one chloride ion) [17]. Recent observations in our laboratory have shown that also tezacaftor (VX-661) and Corr4A rescued p.F508del-CFTR show an increased bicarbonate permeability, similar to that found for lumacaftor [18].

We conclude that the application of bicarbonate is a strategy that could be easily translated in a treatment for CF patients; in fact, the direct treatment with nebulized bicarbonate in CF patients increases the pH and reduces the viscosity of the sputum [21,22,23]. Noteworthy, the bicarbonate therapy may represent a mutation-independent treatment to clear the airways of thickened mucus and improve lung function. The effect of a bicarbonate administration [≥90 mM] in the airways could have a consequence, on the reduction of the pulmonary obstruction, on the increase of the local pH, as well as reduce the barrier effect in the dissolution of the drugs, at the same time. In addition, there is strong evidence about the safety of the use of aerosol bicarbonate, showing that it has no effect on the smooth muscles of the airways [44]. It is therefore an interesting possibility to design a supportive treatment based on topical application of bicarbonate, or any other alkaline buffer, to reduce the viscosity of the airway mucus, and to improve the patients conditions. Indeed, this alkaline treatment could be administered to patients carried of p.F508del-CFTR mutation together with modulators of CFTR activity and to CF patients with other not pharmacologically treatable CFTR mutations on order to ameliorate the respiratory phenotype associated to the CF disease [21]. Moreover, the ability of bicarbonate or other alkaline buffers to reduce mucus viscosity could also be used for other respiratory diseases associated with mucus build-up, such as chronic obstructive pulmonary disease (COPD) in which shortness of breath is associated with recurrent infections such as cystic fibrosis.

## Data Availability

The data presented in this study are available on request from the corresponding author.
